# Comparative Analysis of Various Antioxidants for Rehabilitating the Shear Bond Strength of Composite Resin to Bleached Enamel: An In Vitro Study

**DOI:** 10.7759/cureus.117348

**Published:** 2026-10-02

**Authors:** Ravali Gurijala, Swathi Aravelli, Sivaram Penigalapati, S. Srija Mili, Swetha Kasam, Nimeshika Ramachandruni

**Affiliations:** 1 Department of Conservative Dentistry and Endodontics, Malla Reddy Dental College for Women, Hyderabad, IND

**Keywords:** bleaching, carvedilol, myricetin, quercetin, shear bond strength

## Abstract

Aim: The aim was to evaluate and compare the impact of applying quercetin, myricetin, and carvedilol after bleaching on the shear bond strength (SBS) of composite resin to enamel.

Methods and materials: In this in vitro study, 50 intact single-rooted teeth were randomly allocated into five experimental groups as follows (n = 10): Group A (negative control): no bleaching, Group B (positive control): bleaching with 37.5% hydrogen peroxide, Group C: hydrogen peroxide ± 1% quercetin for 10 minutes, Group D: hydrogen peroxide ± 2.5% carvedilol for 10 minutes, and Group E: hydrogen peroxide ± 1% myricetin for 10 minutes. Bonding with resin composite was carried out without delay following bleaching for Groups B through E, whereas Group A underwent bonding without any prior treatment. Each specimen was assessed for SBS using a universal testing machine after being stored for 24 hours in distilled water kept at room temperature. Tukey's post hoc test and one-way analysis of variance were used to evaluate the SBS values for each group.

Results: The unbleached group showed the greatest mean SBS for composite bonding, according to the data. Quercetin showed the lowest SBS among the antioxidant-treated groups; the carvedilol-treated group showed the highest SBS value, and the difference in SBS between the myricetin and carvedilol groups was not statistically significant.

Conclusions: It was concluded from the data that a 10-minute application of 2.5% carvedilol improved the bond strength to bleached enamel, nearly restoring it to pre-bleaching levels.

## Introduction

Tooth discoloration is a considerable aesthetic concern in the field of dentistry, resulting from a variety of extrinsic and intrinsic factors. Tobacco use, dental plaque, bacterial accumulation, various foods and beverages, and certain medications are typical sources of extrinsic dental staining. On the other hand, dental staining of intrinsic origin primarily results from dental caries, certain medications, trauma, genetics, and aging. Dental prophylaxis could usually eliminate extrinsic stains. If tooth discoloration persists following the removal of external stains, superficial tooth whitening may be recommended [1].

Bleaching materials typically consist of carbamide peroxide and hydrogen peroxide (H_2_O_2_), both of which have been demonstrated to be effective and are widely utilized [2]. Dental restorative materials can have adverse effects, including pulp sensitivity, release of certain components, and changes to the enamel surface [3].

One of the key issues with bleaching materials is the weakened adhesion strength of composite resin and enamel following the bleaching treatment. Approaches to counteract the reduced bond strength comprise application of alcohol to the enamel, eliminating the outer enamel layer, applying organic solvent-based adhesives, and using antioxidant agents [4].

Reports have noted weakening of the bond on both internal and superficial levels, which might be attributed to the existence of residual peroxide. This residual peroxide can interfere with the creation of resinous tags and inhibit resin polymerization [5]. This decrease in bond strength can be prevented by delaying restorative treatments for a duration ranging between one day and three weeks following tooth bleaching, which is a generally used strategy in clinical practice [6]. During this time, residual peroxides are gradually released.

However, there are times when it is necessary to shorten this waiting period due to factors such as patients living far away or having limited time before special events. As a result, researchers are exploring materials that can scavenge free radicals to stop their interference with the bonding process. In this regard, it is advised to use a variety of antioxidants after bleaching, along with prior restoration, such as proanthocyanidin, vitamin E, and 10% sodium ascorbate.

Flavonoids like quercetin, which are present in various fruits, vegetables, and other foods, as well as available as dietary supplements, have been linked to several health benefits, including anticancer, anti-inflammatory, and antioxidant effects [7]. Quercetin could potentially be used extensively to diminish the detrimental impact of bleach on the shear bond strength (SBS) of restorations.

Carvedilol is a medication that offers antioxidant as well as anti-inflammatory effects [8]. Being a non-selective β-adrenergic blocker, it is commonly prescribed for the management of hypertension, congestive heart failure, and coronary artery disease [9]. Additionally, its cardioprotective, nephroprotective, and hepatoprotective effects have been well established. Another intriguing finding is that carvedilol administration in diabetic animals exhibited a reduction in diabetes-related bone disease, likely due to its antioxidant properties [10].

It has also been shown that treating rats with carvedilol reduces the inflammatory response in periodontal inflammation by lowering the levels of proinflammatory cytokines such as interleukin-1β (IL-1β) and tumor necrosis factor-α (TNF-α), as well as cyclooxygenase-2 (COX-2), a crucial enzyme frequently found in inflammatory tissues [11].

Natural polyphenols like myricetin [12], resveratrol, and kaempferol possess multiple phenolic -OH groups that establish hydrogen bonds with the collagen’s amide carbonyl groups. This interaction helps to alter the collagen structure and inhibit matrix metalloproteinases (MMPs) [13]. Additionally, the free -OH groups can bind to metal cations (such as Ca^2+^, Mg^2+^, and Au^2+^), which may enhance the deposition of amorphous calcium phosphate (ACP) nanoparticles and promote the formation of hydroxyapatite (HA) [14].

This study aims to assess and compare the impacts of quercetin, myricetin, and carvedilol in reversing the decrease in composite resin's SBS to bleached enamel. The null hypothesis asserts that the use of antioxidant solutions has no impact on SBS.

## Materials and methods

Specimen preparation

The current in vitro study was conducted following the established guidelines of the Institutional Ethics Committee and was approved by the same (MRDCW/IEC/AP/24/2024); 50 extracted intact single-rooted teeth (Figure 1) were collected. Power analysis was performed using G*Power (Version 3.1; Heinrich-Heine-Universität Düsseldorf, Düsseldorf, Germany). Based on the effect size derived from previous similar studies, with a significance level of 0.05 and a statistical power of 80%, a minimum sample size of 10 specimens per group was determined to be sufficient to detect significant differences in SBS among the groups. The study followed a single-blind design. The evaluator responsible for measuring SBS was blinded to the group allocation to minimize assessment bias.

**Figure 1:**
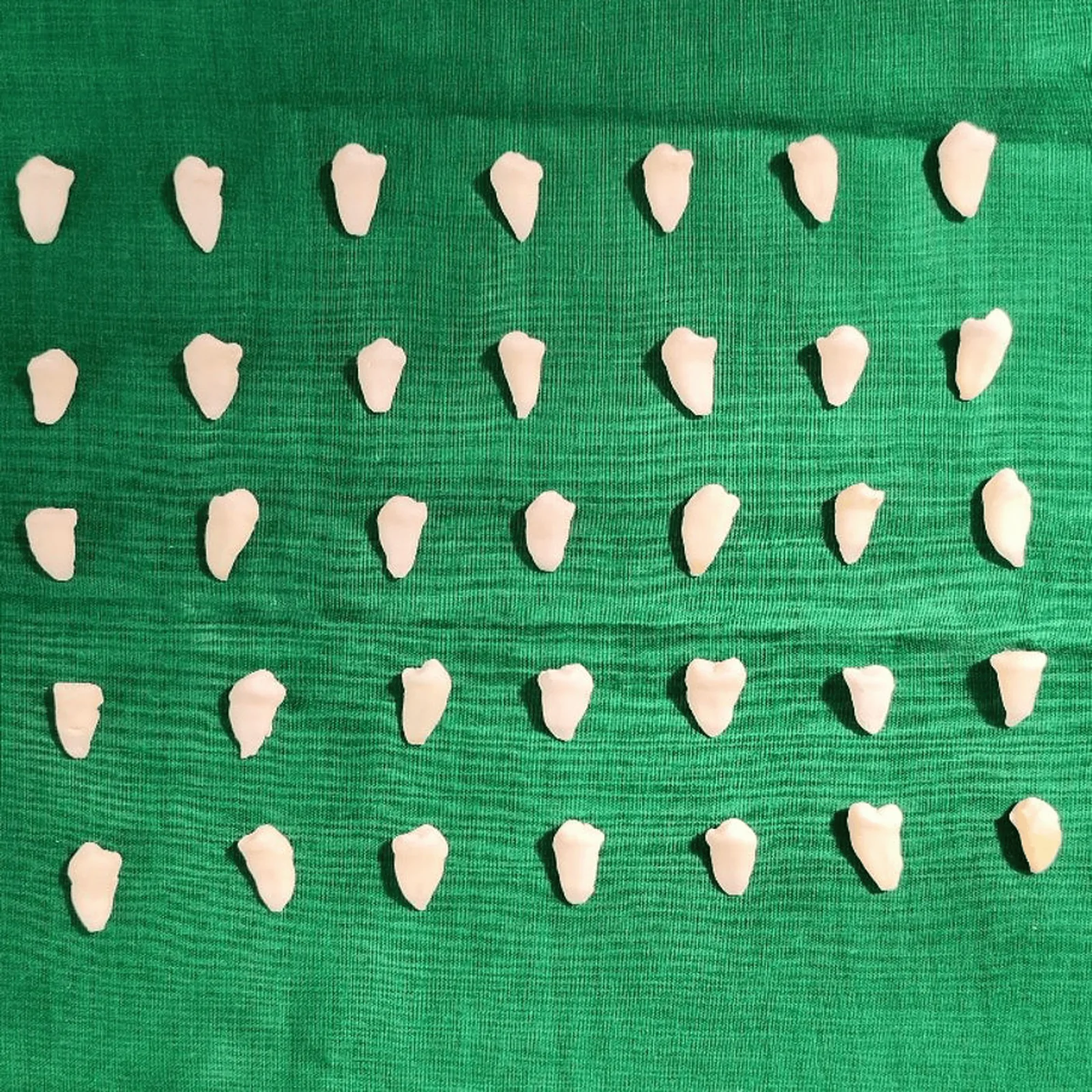
Extracted teeth

The extracted teeth were preserved in 0.5% chloramine solution at 4 degrees Celsius for no longer than two months prior to use. Each tooth was then embedded in cylindrical acrylic resin blocks of 2 cm in diameter and 2.5 cm in height, with the cementoenamel junction (CEJ) positioned at the base of the block, leaving the coronal segments of the teeth exposed.

Ensuring continuous application of water, the enamel surfaces were polished using silicon carbide (600-grit) paper mounted on a low-speed handpiece [14]. The enamel surfaces of 50 teeth were then bleached using a 37.5% hydrogen peroxide gel, applied twice for 20 minutes each, following the instructions of the manufacturer. After bleaching, tap water was used thoroughly to rinse off the gel.

Treatments

A total of 10 teeth that hadn’t been bleached were assigned to Group A (negative control), while the remaining teeth were allocated to four groups at random (n = 10 each). The allocation of samples into different groups was performed using a simple randomization method. Each specimen was assigned a number, and group allocation was carried out using a computer-generated random sequence to ensure unbiased distribution.

In Group B (positive control), the teeth had undergone bleaching without the application of antioxidants. In Group C, 1% quercetin solution (Figure 2) was formulated by dissolving 1 g of quercetin powder (Sigma-Aldrich, St. Louis, MO, USA) [15] in a standard flask containing 100 mL of ethyl alcohol and heating in a water bath at 37°C (Figure 3).

**Figure 2:**
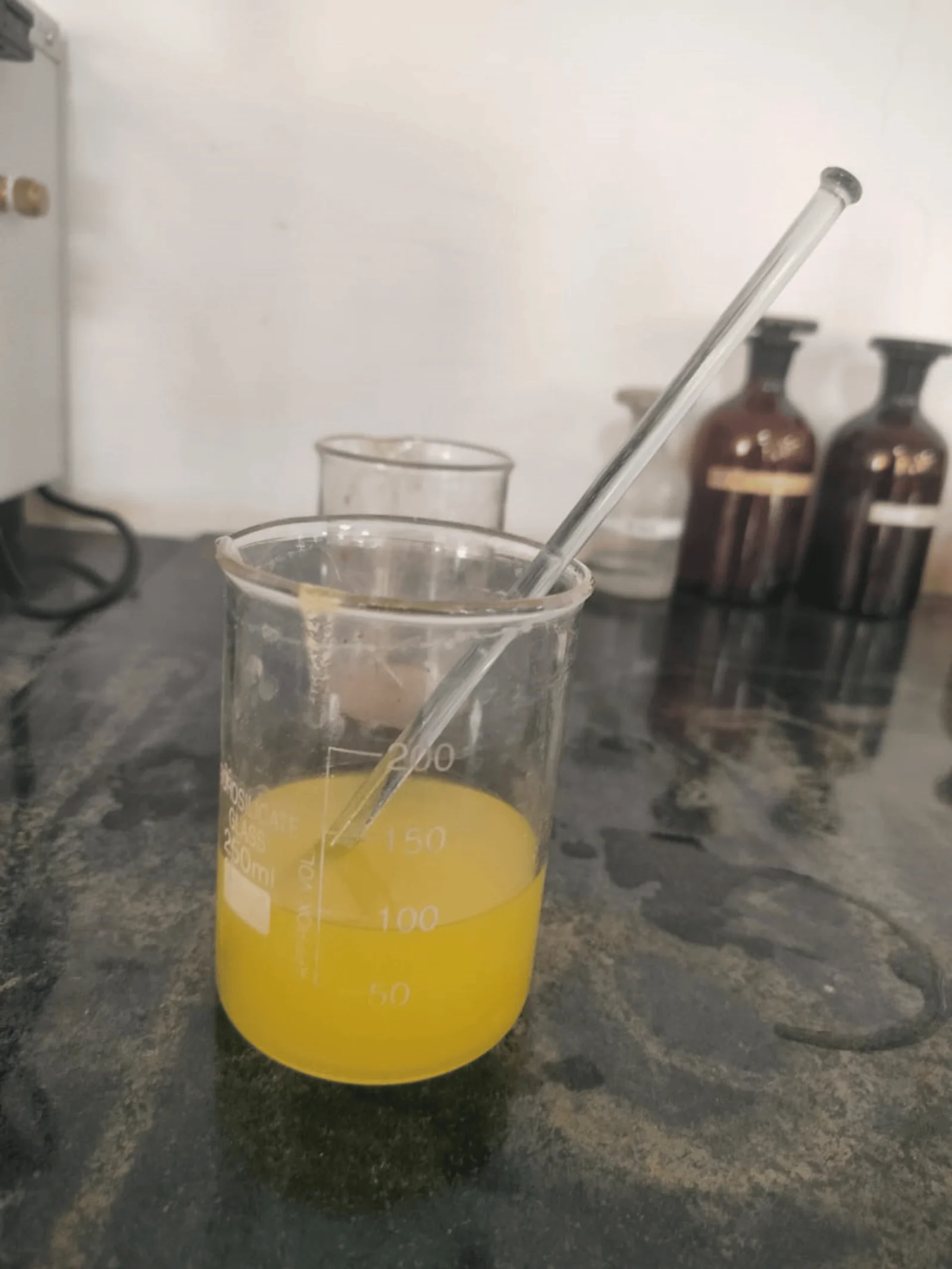
Quercetin solution

**Figure 3:**
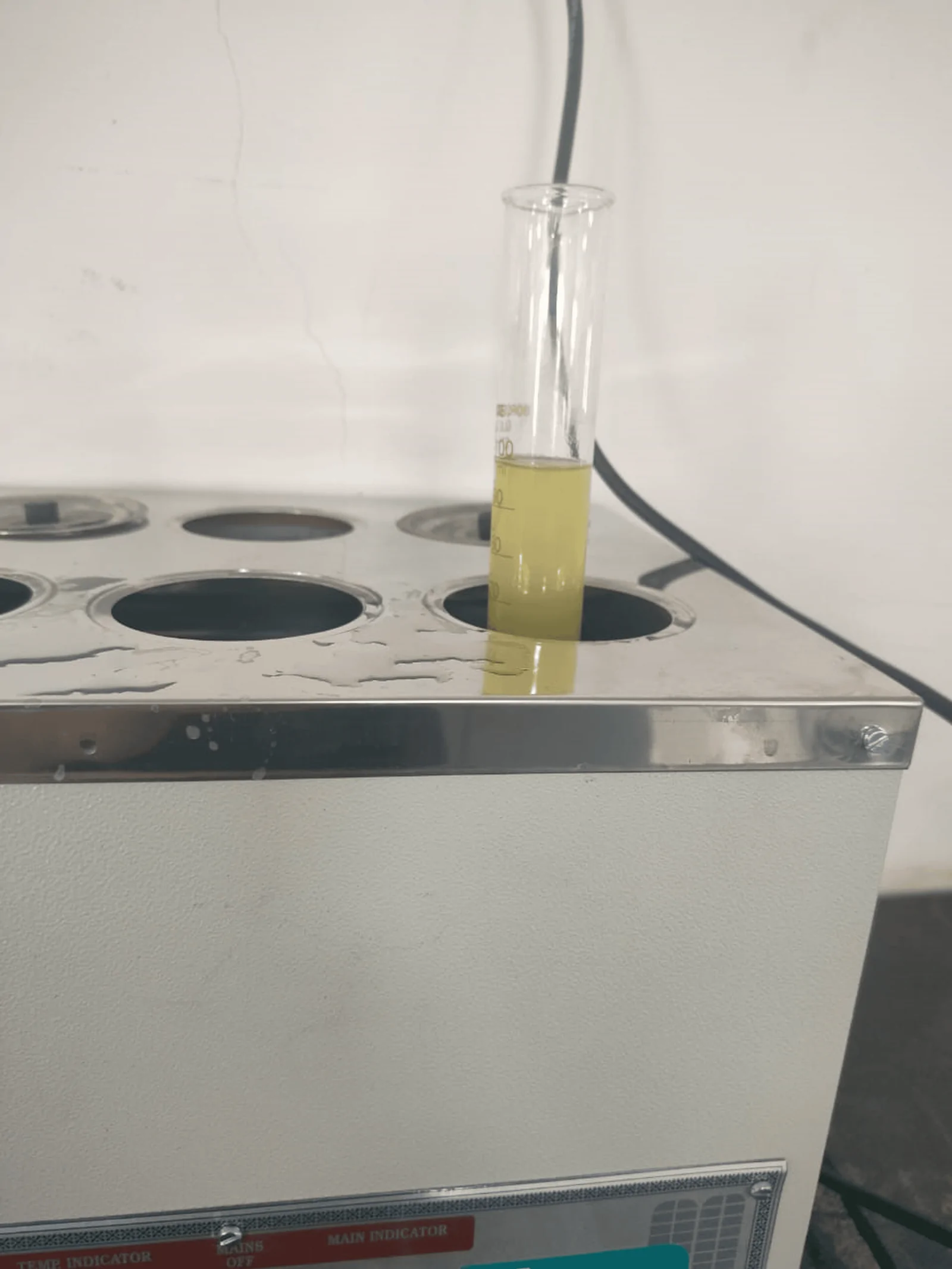
Quercetin solution heating at 37°C

In Group D, 2.5% carvedilol solution [11] (Figure 4) was formulated by dissolving 2.5 g of carvedilol powder directly into saline.

**Figure 4:**
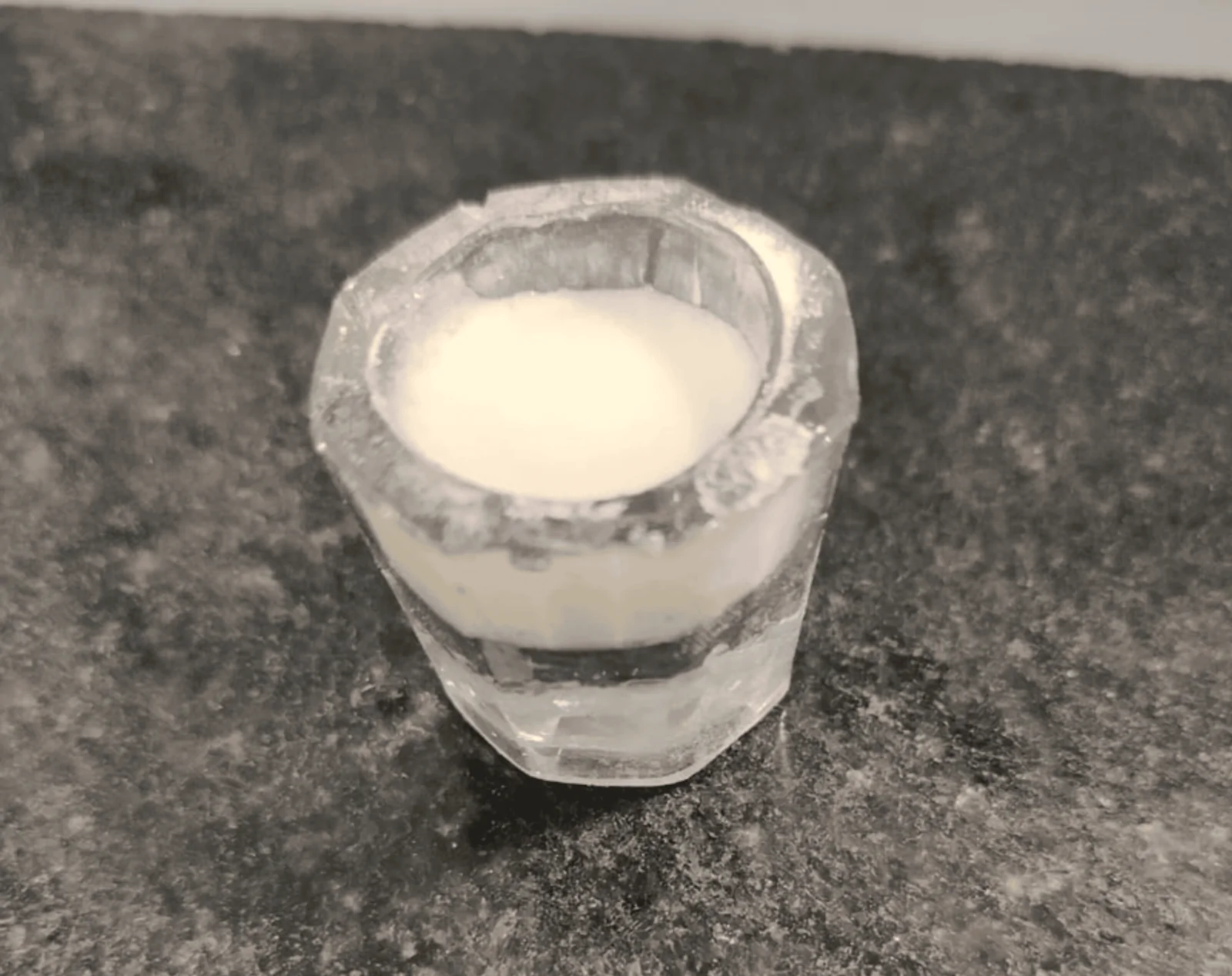
Carvedilol solution

In Group E, 1% myricetin solution was formulated by dissolving 1 g of myricetin powder (Sigma-Aldrich) in 100 mL of pure ethanol and heating it in a water bath at 37°C (Figure 5) [13].

**Figure 5:**
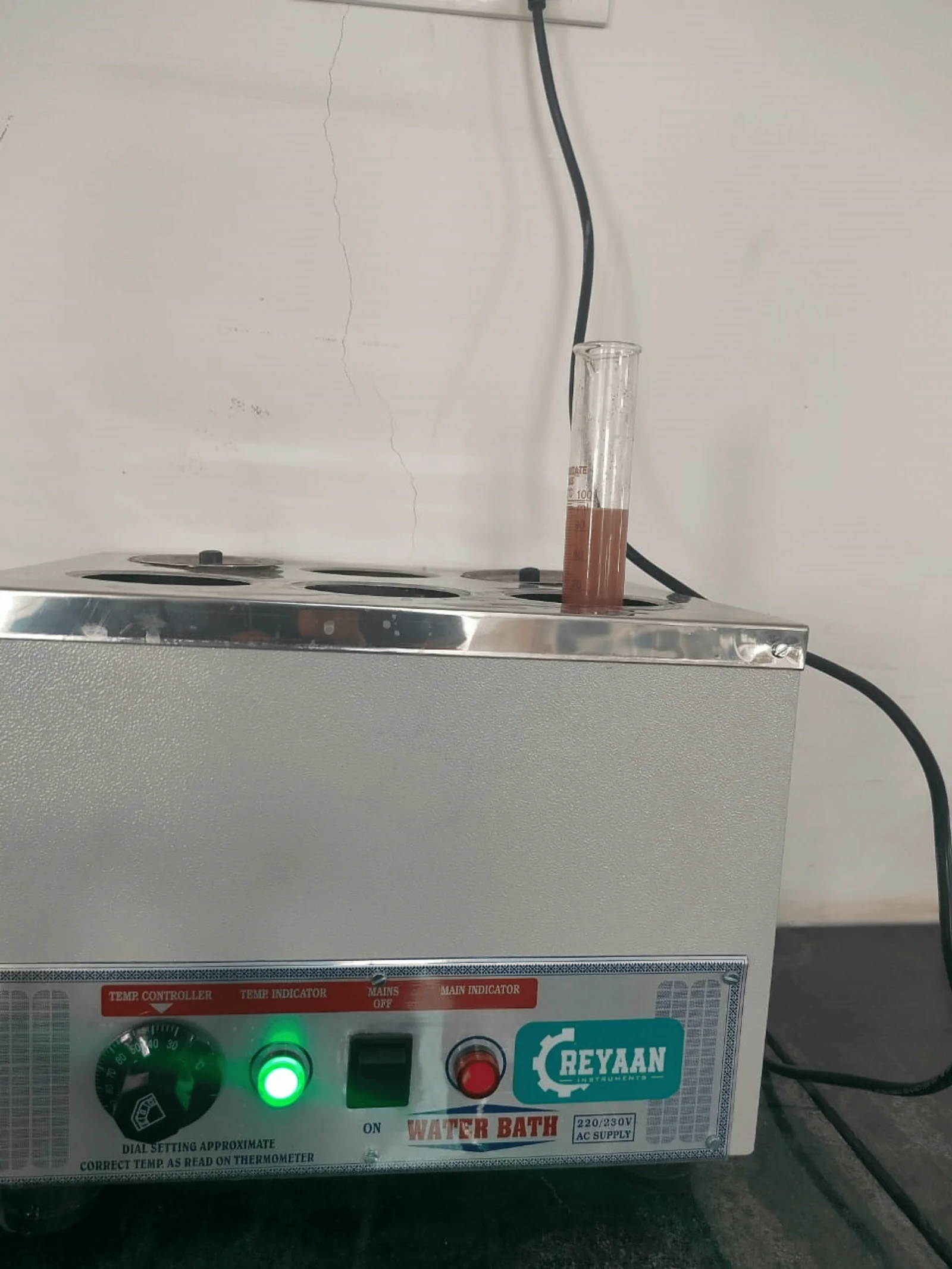
Myricetin solution heating at 37°C

Following the bleaching procedure, the respective antioxidants - quercetin solution [16] (Figure 6), myricetin solution (Figure 7), and carvedilol solution (Figure 8) - were applied to the enamel surfaces for 10 minutes.

**Figure 6:**
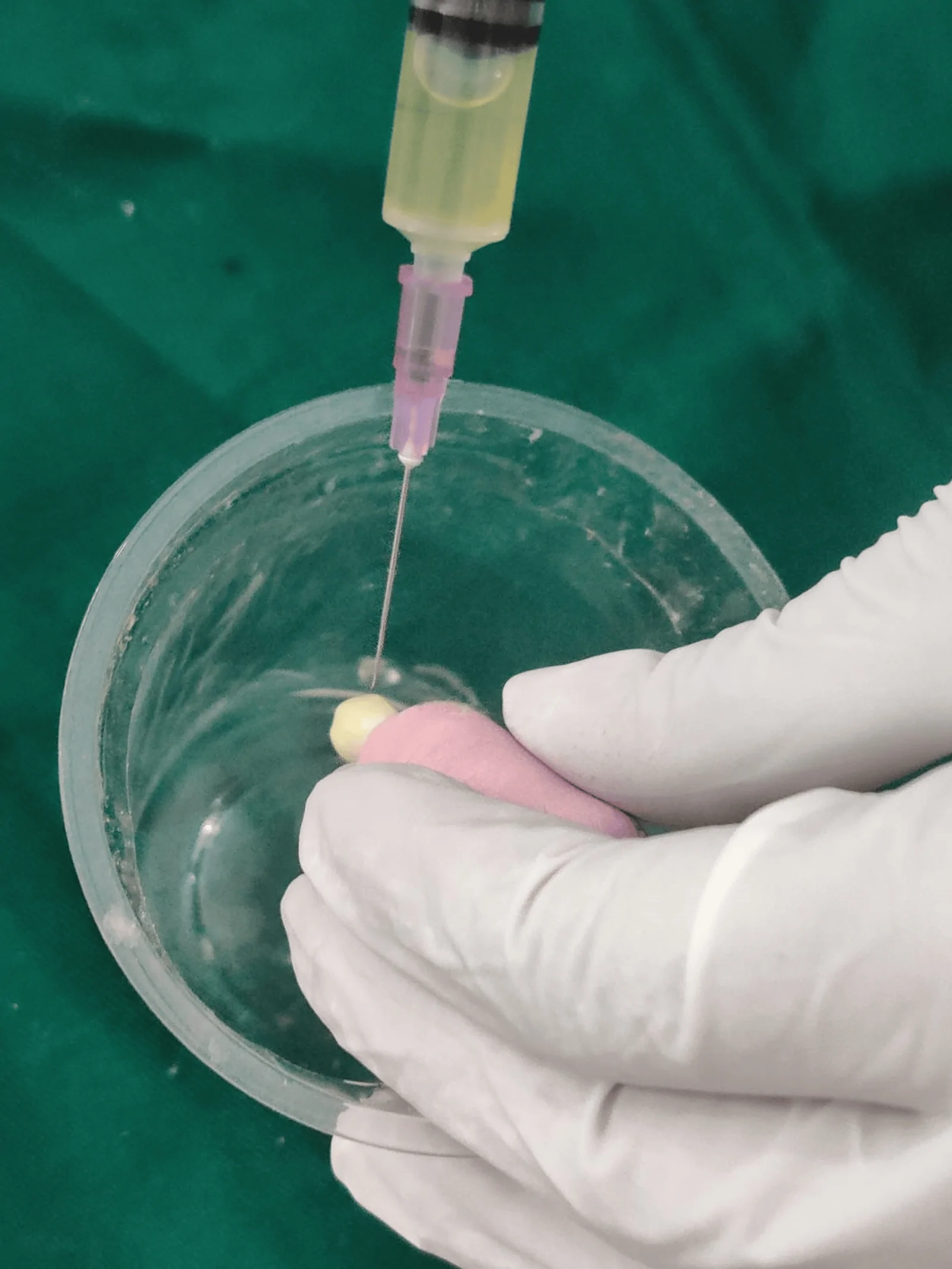
Quercetin solution application

**Figure 7:**
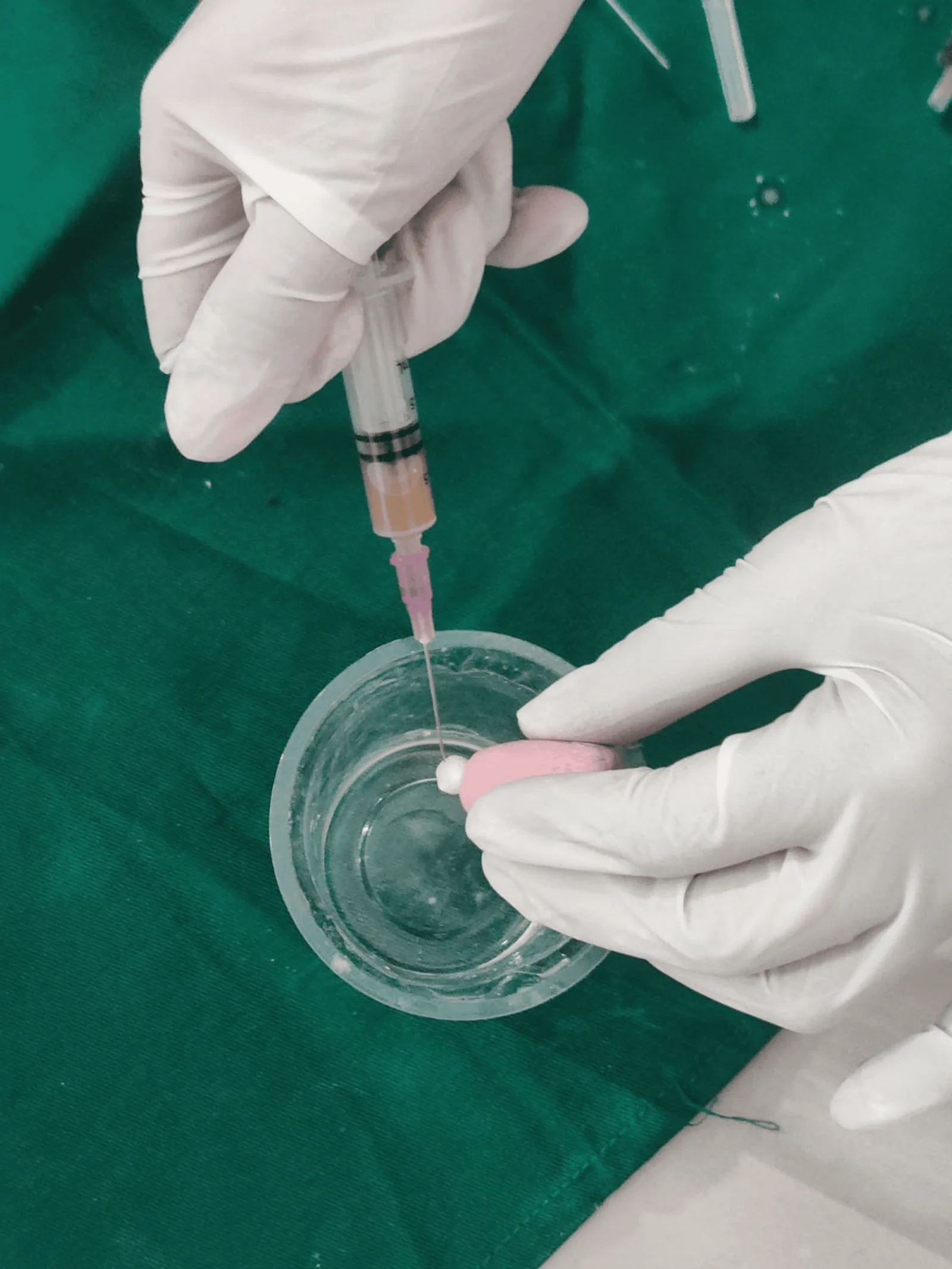
Myricetin solution application

**Figure 8:**
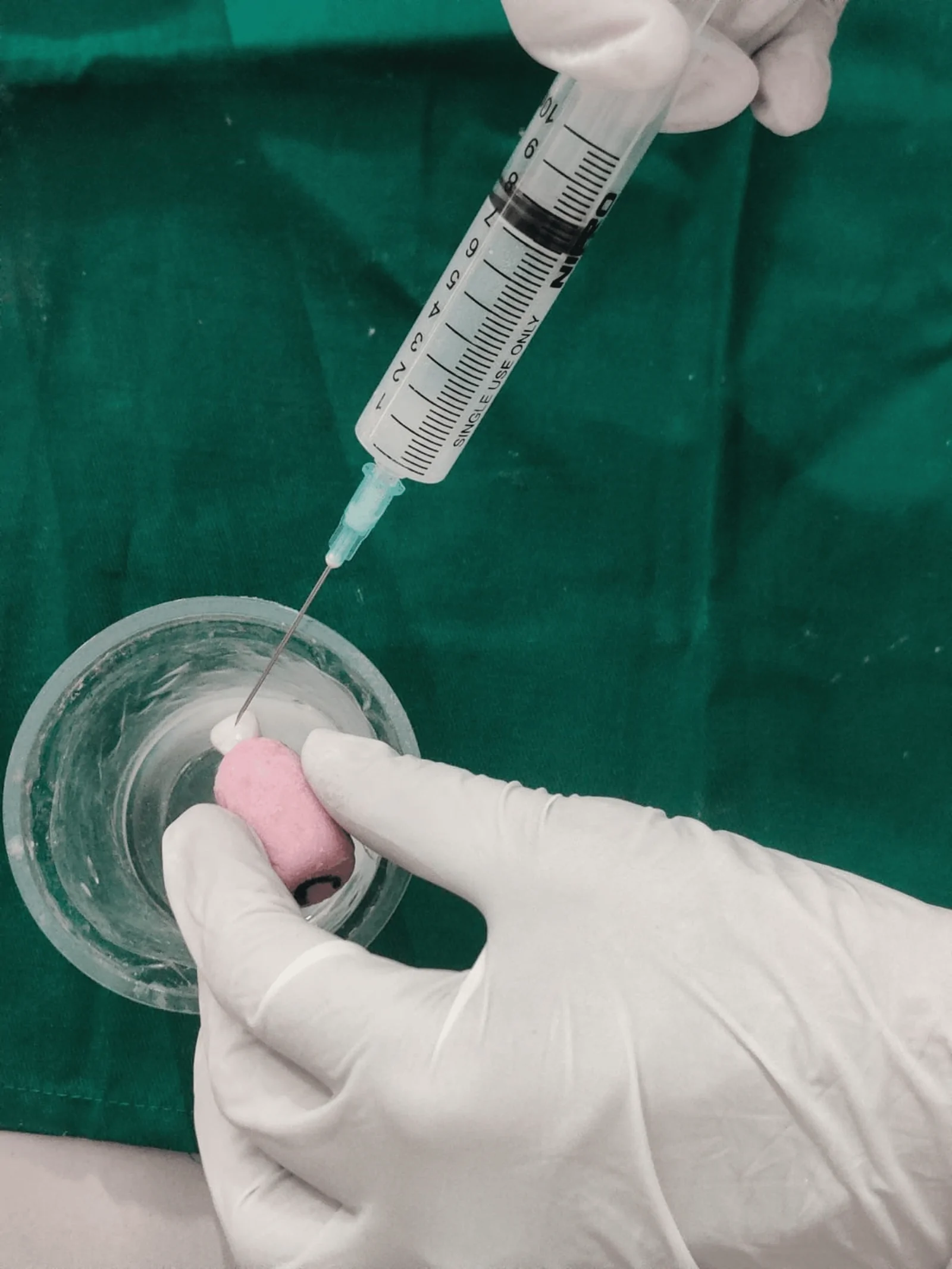
Carvedilol solution application

The specimen received the solution via syringe every minute, with additional refreshing by a microbrush to maintain the enamel surface in a moist state. A 30-second water rinse was performed on the specimens, followed by air-drying.

Restorative procedure

Etching of the bleached surfaces was done with 37% phosphoric acid for about 20 seconds, followed by a water rinse for another 20 seconds. The samples were then treated with Adper Single Bond (3M ESPE, St. Paul, MN, USA), which was light-cured for 20 seconds using an LED device with a light intensity of 1200 w/cm^2^. With the aid of a plastic mold that was 3 mm in diameter and 2 mm in height, a composite resin restoration (Tetric N-Ceram, Ivoclar, Schaan, Liechtenstein) (Figure 9) was done, followed by curing for 20 seconds. Immediately after, the specimens were stored in distilled water at room temperature for the next 24 hours.

**Figure 9:**
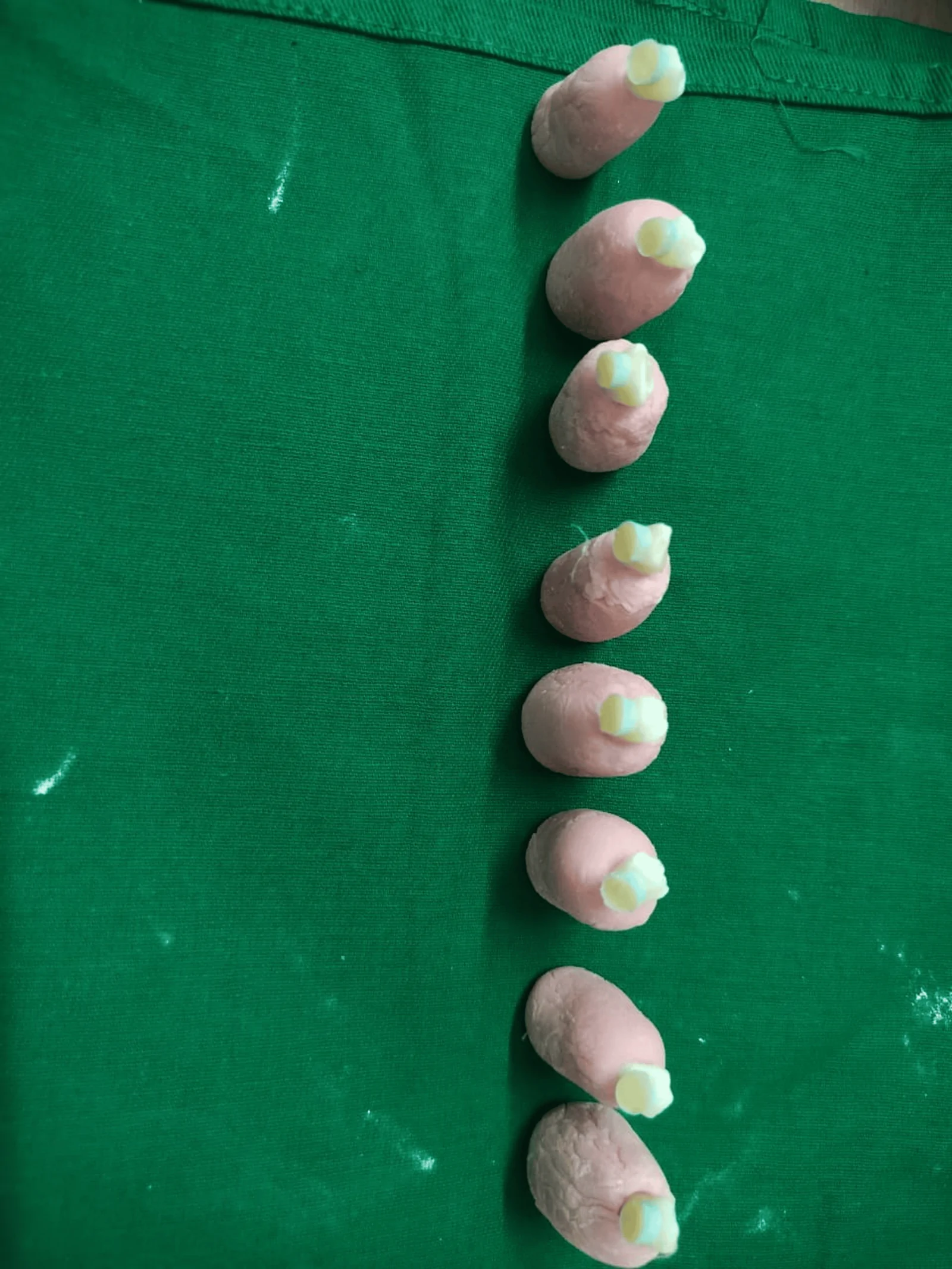
Composite resin restoration bonded to the tooth surface

SBS testing

Shear testing was performed on the specimens with a chisel-shaped blade mounted on a universal testing machine (Figure 10), with a crosshead velocity set at 1 mm/min.

**Figure 10:**
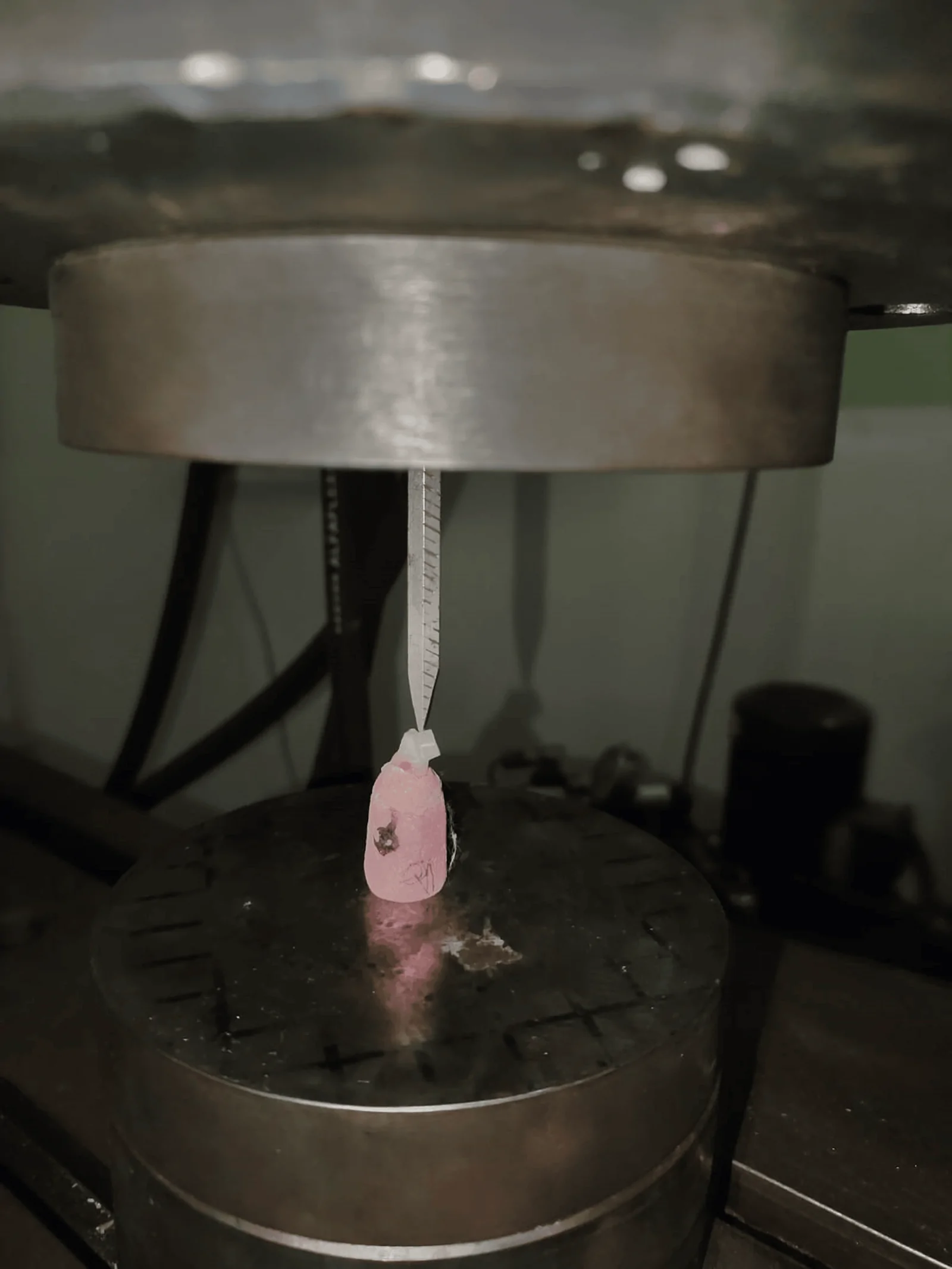
Universal testing machine

Failure force values were measured in Newtons (N), and the SBS measurements were quantified in megapascals (MPa). Then, the SBS in MPa was determined using the equation outlined below:

$\text{Bond strength (MPa)} = \frac{\text{Peak force (Newtons)}}{\text{Area (m}^2\mathrm{)}}$

Statistical analysis

The data that were collected were evaluated with the help of IBM SPSS Statistics for Windows, Version 25 (Released 2017; IBM Corp., Armonk, New York, United States). Shapiro-Wilk test was employed to analyse the data normality. Next, a one-way analysis of variance (ANOVA) was used to compare the groups studied based on SBS.

## Results

The SBS comparison among the five study groups revealed a statistically significant difference, as shown by the one-way ANOVA test (p < 0.001). This reveals that the type of surface treatment had a significant impact on the resulting SBS values.

Groupwise mean SBS values

The mean SBS and standard deviation values of each group are presented in Table 1. The unbleached enamel group (Group A - negative control) demonstrated the highest bond strength value (22.68 ± 2.91 MPa), establishing it as the reference point for optimal bonding conditions unaffected by bleaching.

**Table 1:** Mean and standard deviation of shear bond strength (SBS) (MPa) in each group

Groups	Mean (SBS ± SD)
Group A (negative control)	22.68 ± 2.91
Group B (positive control)	12.29 ± 2.20
Group C (quercetin)	15.92 ± 2.72
Group D (carvedilol))	21.11 ± 2.20
Group E (myricetin)	20.12 ± 2.92

In contrast, the bleached enamel group without antioxidant treatment (Group B - positive control) showed the lowest mean SBS (12.29 ± 2.20 MPa). This substantial reduction highlights the detrimental effect of bleaching on the immediate bonding ability of composite resin.

Effect of antioxidant application

Among the antioxidant-treated groups, improvements in SBS were observed when compared to the positive control.

Quercetin-treated samples (Group C) demonstrated a moderate increase in SBS (15.92 ± 2.72 MPa), showing partial reversal of the bleaching-induced reduction. Carvedilol (Group D) exhibited the highest SBS among the antioxidant groups (21.11 ± 2.20 MPa), approaching values that are almost comparable to the unbleached group. Myricetin (Group E) also increased SBS, with a mean SBS of 20.12 ± 2.92 MPa.

Although carvedilol showed the highest recovery of bond strength, the difference in SBS between the myricetin and carvedilol groups was not statistically significant, indicating comparable efficacy between the two antioxidants.

Graphical representation

Figure 11 depicts a bar graph comparing the mean SBS ± SD across all groups. It clearly highlights the statistically significant improvement in SBS after antioxidant application, with carvedilol and myricetin showing the closest recovery of SBS values to unbleached enamel bond strength when compared to quercetin.

**Figure 11:**
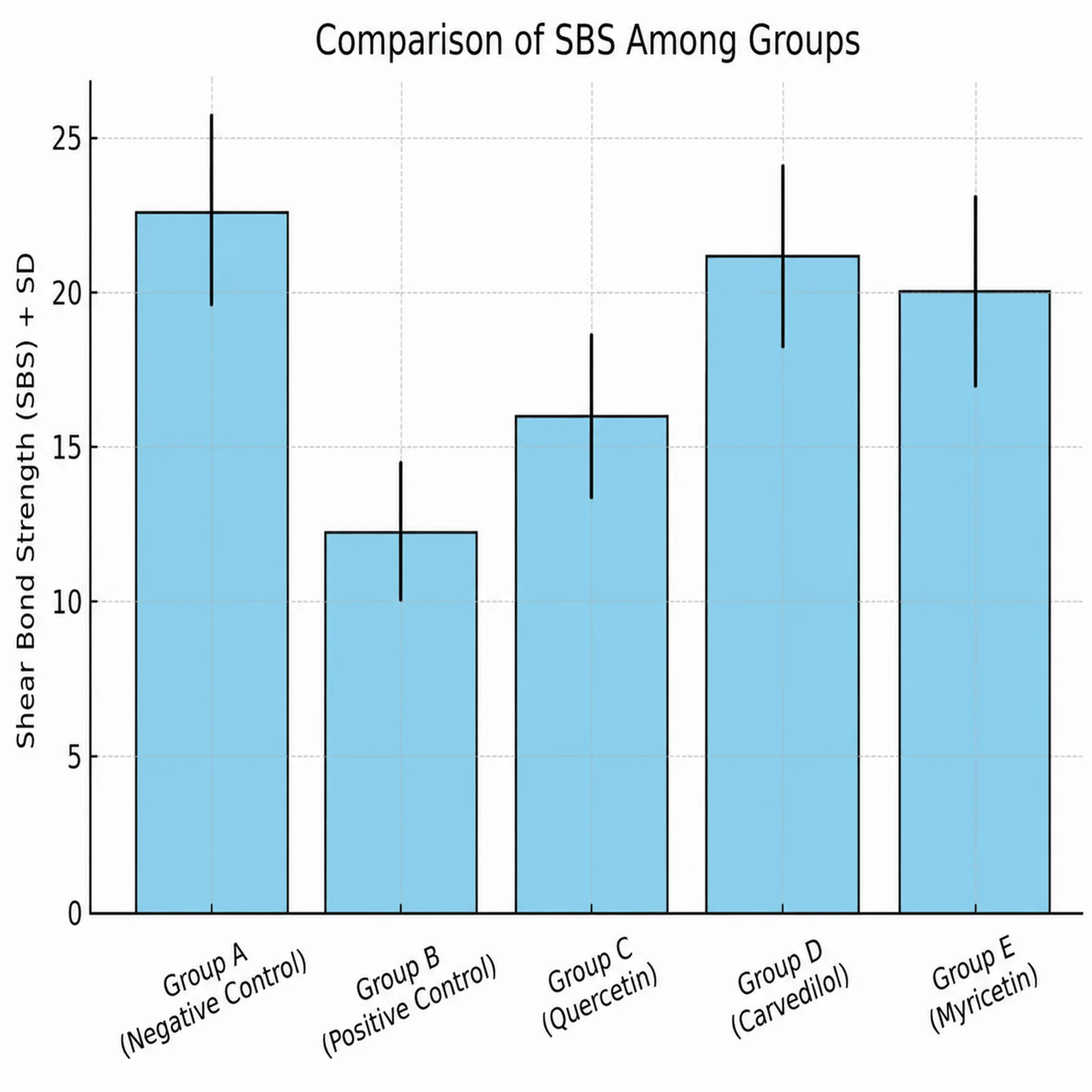
Bar graph comparing the mean shear bond strength (SBS) values (±SD) among all groups

## Discussion

Antioxidants have been identified as the most effective, rapid, and convenient method for re-establishing bond strength, which was compromised by bleaching [16]. The results demonstrated a significant improvement in SBS following bleaching. Application of quercetin, carvedilol, and myricetin increased bond strength compared with the bleached control group. Among the tested antioxidants, carvedilol and myricetin showed the highest bond strength values, approaching those of the unbleached control group. These findings suggest that carvedilol and myricetin were effective in reversing the adverse effects of bleaching on enamel bond strength.

The null hypothesis was rejected, as carvedilol and myricetin successfully restored the bond strength of bleached enamel to levels comparable with the unbleached control. Due to prior studies showing that the enamel exposed to 10 minutes of quercetin treatment had the best results, quercetin had been chosen as the gold standard in this investigation. Following bleaching, a 1% quercetin solution can rapidly restore the SBS. Because of its positive qualities, such as preventing the generation of reactive oxygen species (ROS), quercetin is seen as a potential substance for increasing the bond's longevity. Additionally, it acts as an antimicrobial substance that works against both ROS and COX-2, which subsequently aids in lowering the activity of MMPs [16], viruses, as well as Gram-positive and Gram-negative bacteria [17].

Due to the above properties, quercetin can serve as an important compound in the prevention of recurrent caries. Release of quercetin occurs in two steps: (1) the burst phase, where antioxidants are released in larger quantities during the early phase, and (2) the tail release phase, where a low level of antioxidant release takes place that falls below the effective level, which happens after a few weeks. However, quercetin has poor solubility, which is believed to prevent it from being dissolved by saliva, making it well suited for use as a sustained antioxidant release material [17]. Additionally, quercetin remains beneficial due to its high antioxidant activity up to two weeks post-bleaching. Some studies have demonstrated its effectiveness in preventing the degradation of bonds at the dentin-resin interface [16].

There were few studies that have explored the impact of quercetin on reversing the SBS following bleaching. An earlier study by Shamsedin et al. (2017) proved that various concentrations of quercetin (0.1%, 0.5%, and 1%) were effective in improving the values of SBS to baseline levels, irrespective of application time (5 and 10 min), in support of outcomes observed in the current study. Nevertheless, although quercetin showed an increase in SBS in the present investigation, the SBS values were comparatively lower than those of myricetin and carvedilol.

In the present study, the application of 2.5% carvedilol solution for 10 min helped in reversing SBS; the improvement in bond strength was significant when it was compared to the positive control group and quercetin group. To the best of our knowledge, this was the first study that evaluated the antioxidant effect post-bleaching.

According to the literature, carvedilol possesses antioxidative and anti-inflammatory properties [8]. It helps in lowering pro-inflammatory cytokines, accompanied by a rise in anti-inflammatory cytokines, which is responsible for its anti-inflammatory action. Glutathione, a protein possessing antioxidant potential, is responsible for its antioxidant action, subsequent to the carvedilol use during lipid peroxidation [18]. Many studies have shown that it alleviates diabetes in rodents, as well as associated bony lesions. Thus, these features suggest that the product may help in reducing the oxidative stress caused by the H_2_O_2_ of the bleaching gel.

In the previous study, after 30 days, it was noted that the pulp tissue of molars treated with bleaching gel remained well-organized and free from inflammation. This finding aligns with earlier studies, where, despite extensive pulp injury caused by the bleaching procedure, the pulp tissue could restore its homeostasis following this period [19], in diabetic rats as well. Additionally, in another study, it was noted that cell proliferation in the dental pulp followed bleaching, which was followed by subsequent tissue organization [20]. These observations could help in explaining the reduction in dental sensitivity reported by patients after some time following the bleaching procedure.

Better antioxidant activity of carvedilol than quercetin might be due to its higher percentage, which is of 2.5. Further studies need to be conducted to assess the antioxidant effects of carvedilol post-bleaching. The findings demonstrated that a 1% myricetin solution, when applied for a duration of 10 minutes, could promptly assist in restoring the SBS after bleaching. This enhancement was statistically significant when compared to the positive control group. Nevertheless, the statistical analysis indicated no notable differences between the carvedilol and myricetin groups.

Myricetin, a natural polyphenol containing several phenolic -OH groups, interacts via hydrogen bonding with the amide carbonyls in collagen. This interaction helps modify the collagen structure and inhibits the activity of MMPs [21]. When comparing myricetin and quercetin as antioxidants, particularly in post-bleaching dental treatments, both are flavonoids known for their strong free-radical scavenging abilities, but they differ in potency, structure, and potential clinical outcomes. Myricetin has six hydroxyl (-OH) groups on its flavonoid backbone, whereas quercetin has five hydroxyl (-OH) groups; more hydroxyl groups typically mean higher antioxidant potential, since they donate hydrogen atoms to neutralize free radicals.

Myricetin generally exhibits stronger antioxidant capacity than quercetin in vitro models, due to its additional hydroxyl group, which enhances radical scavenging and metal chelation properties. Quercetin is still a potent antioxidant but may be slightly less effective than myricetin under oxidative stress conditions [22]. Quercetin has been studied more extensively than myricetin in dental applications. Further research has to be done to evaluate the effects of myricetin.

This study has certain limitations. As an in vitro investigation, it does not fully replicate the complex oral environment, including saliva, temperature fluctuations, and masticatory forces. Additionally, only the immediate SBS was evaluated, and the long-term durability of the bond was not assessed. Therefore, the findings should be interpreted with caution when extrapolating to clinical practice. Further in vivo and long-term studies are recommended to validate the effectiveness of the tested antioxidants under actual clinical conditions.

## Conclusions

According to the results of this study, bleaching markedly reduced the SBS between resin composite and bleached enamel. Within the limitations of the study, it showed that carvedilol and myricetin exhibited greater antioxidant activity post-bleaching than quercetin. Further research efforts are required to evaluate the antioxidant potential of myricetin and carvedilol post-bleaching.
